# Thermoset Polymer Matrix Composites of Epoxy, Unsaturated Polyester, and Novolac Resin Embedding Construction and Demolition Wastes powder: A Comparative Study

**DOI:** 10.3390/polym13050737

**Published:** 2021-02-27

**Authors:** Costas Bogiatzidis, Loukas Zoumpoulakis

**Affiliations:** Laboratory Unit of Advanced and Composite Materials, School of Chemical Engineering, National Technical University of Athens, 9-Heroon Polytechniou str., Zografou Campus, 15773 Athens, Greece; lzoubou@chemeng.ntua.gr

**Keywords:** polymer-matrix composites, mechanical properties, thermal properties, materials characterization, construction and demolition wastes

## Abstract

Composite materials that consisted of a polymer resin as matrix (PMCs), filled using construction and demolition (C&D) wastes powder of different grain sizing in micro-scale were manufactured and studied. Three different kinds of resins were used as the matrix for the purposes of this study. More specifically, composites made of epoxy and unsaturated polyester resins purchased from the market and phenolic resin (novolac) laboratory synthesized, were produced. The morphological and elemental analysis of these materials was performed through scanning electron microscopy (SEM), energy dispersive X-ray spectroscopy (EDX), and X-ray diffraction (XRD). Additionally, mechanical performance and thermal insulating efficiency of these materials were examined through bending and shear strength tests according to the three-point method and via determination of the thermal conductivity coefficient *λ.* C&D wastes have undergone the appropriate processing in order to prepare filling products of the required granular size in pulverized form. In this research study, construction and demolition waste-based thermosetting polymer composites were produced with flexural strength in the range 20–60 MPa, shear strength in between 1–8 MPa, and thermal conductivity coefficients in the range of 0.27–1.20 W/mK. The developed materials embedded 30–50% *w*/*w* C&D wastes, depending on the resin used as the matrix.

## 1. Introduction

Polymer matrix composites (PMCs) are extensively used in numerous applications nowadays. The low cost, simple manufacturing techniques and the relatively good properties that these materials exhibit, have placed them in a dominating position in many technological and scientific aspects [1,2,3,4,5,6,7,8,9,10,11,12,13].

Moreover, PMCs are also characterized by significantly good heat insulation efficiencies in comparison to conventional materials. This is attributed to the low thermal conductivity coefficient of the polymer system that is usually used to form the PMCs’ matrix and has led to their wide utilization in many applications within the construction sector [14,15,16,17,18,19,20,21,22,23,24].

During the last decades, researchers have focused on the development of materials with enhanced mechanical properties [25,26,27,28,29,30,31,32,33]. The high costs of some reinforcing additives, however, have driven scientific research into the consideration of alternative kinds of filling (embedding additives) materials in composites’ manufacturing [34].

At the same time, environmental awareness issues created the circumstances under which byproducts of various human activities were reviewed and exploited as potential fillers in PMCs’ manufacturing [35,36,37,38,39,40,41,42,43]. On the other hand, the issue concerning the treatment of byproducts deriving from construction, demolition, and renovation projects combined with the good thermo-mechanical characteristics of these materials, has intrigued the interest of scientists. A significant number of research papers in which holistic management strategic plans were introduced in order to optimize the recycling of construction and demolition (C&D) waste stream were published [44,45,46,47,48]. Up until now, however, many countries worldwide have been unable to meet the demands of environmental legislation and achieve the extremely high (in terms of quantities) targets set for the recycling of waste produced from the construction sector [49,50]. As a result, environmentally harmful treatment methods such as landfilling and illegal dumping are still in use in the management of this specific waste stream [51].

The high recovery potential of these materials, which at the moment remains unexploited, has focused the most recent research efforts on the development and study of new composite materials embedding construction and demolition wastes as filler in the form of micro-powder [52,53,54,55,56]. 

In the present study, thermosetting polymer composites of epoxy resin (ER), unsaturated polyester (UP), and novolac resin (N) embedding pulverized C&D waste in micro-scale sizes were developed using the manufacturing techniques described in previously mentioned studies [52,53,56]. The scope of this research is to investigate the appropriateness of this specific kind of filler in composite materials manufacturing through the comparative study of the thermo-mechanical performance of these materials.

The degree of novelty and the significance of this research are very high because the inclusion of C&D micro-particles in thermoset polymers is resulting in the production of materials with adequately good mechanical properties and thermal insulating capacity. In addition, through the manufacturing of these new composites, an innovative way of exploiting the spin-offs of the construction sector, which are produced in very large quantities worldwide each year, is introduced. In terms of treatment and exploitation, the optimum scenario for these materials, if not disposed of in landfills or non-authorized dump sites, is their recovery by means of backfilling. The results of this study will provide useful conclusions on the exploitation possibilities of the development of new building materials with fillers made using wastes of similar categories such as marble mining and processing residues, concrete and cement production industry waste, bricks manufacturing industry waste, etc.

## 2. Materials and Methods

### 2.1. Embedding Substance Preparation

A mixture of C&D waste aggregates generated from the construction, demolition, and renovation sites was collected and appropriately treated as described in previous research papers [52,53]. Fine micro-granular additive material was produced in order to be used as filler. Two different grain size filling powders in flour form were prepared via mechanical splitting and sieving separation processes according to ASTM C 136.

Analytically, C&D waste was dried in a laboratory oven at a constant temperature as recommended per the above standard and was then subjected to sieving by means of manual sieves and a mechanical sieve shaker in order to produce aggregate samples of the desired grain characteristics for the purposes of this research [57].

### 2.2. Polymer Resins Used as Matrices-Specimens’ Manufacturing Process

Three different polymer resin systems were used as matrices in PMCs’ manufacturing. More specifically, composite materials of (i) EPOXOL 2874 two-part epoxy resin (ER) system, a bisphenol-based epoxy that comes with selected chemicals as curing agents such as amines [58,59] (Neotex Co., Attica, Greece); (ii) PE6/TC two-part unsaturated polyester (UP) system (Neotex Co., Attica, Greece); and (iii) Novolac (N) (in-house synthesized, NTUA, Athens, Greece) resin were prepared. Epoxy and polyester resins were purchased from the market. Novolac resin was laboratory-produced through progressive polymerization. The synthesis process of N is based on the poly-condensation reaction of phenol (Merck, Darmstadt, Germany) under the presence of formaldehyde (Fluka, NC, USA) and the appropriate chemical catalyst (acetic acid, Fluka, NC, USA). The resulting polymer belongs in the category of phenol-formaldehydes. It comes initially in solid-state that require pulverization, manual sieving, and addition of hexamethylenetetramine (Merck, Darmstadt, Germany) as a hardener in its curing [60,61,62,63]. The technical specifications of epoxy, unsaturated polyester, and novolac resin systems are presented in Table 1.

The manufacturing techniques implemented for ER, UP, and N composites’ preparation, and the related details (i.e., mixture ingredients, *w*/*w* proportions for each resin, filler-resin *w*/*w* proportions, mixing time, thermal curing and post-curing time, etc.), were presented and analytically discussed in previous research studies [52,53,56]. Table 2 presents the different categories of specimens manufactured and examined within the scope of this research.

### 2.3. Thermo-Mechanical Properties

**Figure 1:**
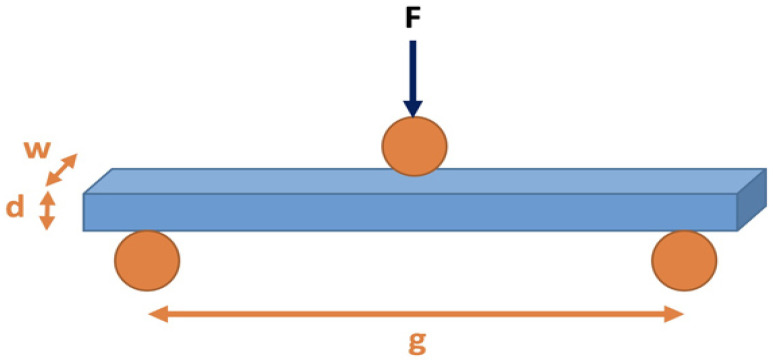
The three-point method set up used for bending and shears properties measurement.

**Figure 2:**
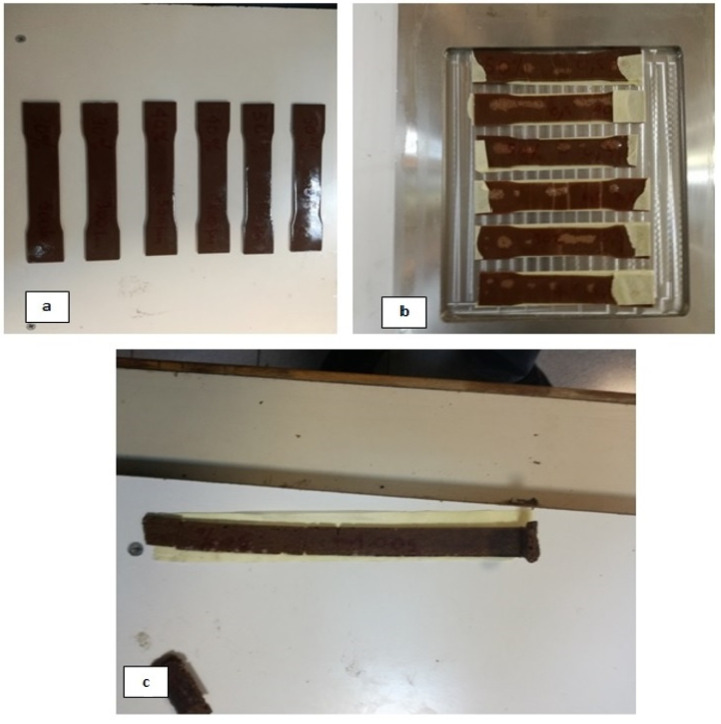
Specimens made for bending and shear strength tests (**a**) unsaturated polyester composites, (**b**) epoxy composites, and (**c**) novolac composites.

Flexural and shear properties tests were carried using a three-point method, in compliance with standards ASTM D 790 and ASTM D 2344, respectively [64,65]. At least five specimens were prepared and experimentally characterized for each different category of polymer composites studied as recommended by testing standards. The distance between the specimen supports of the testing arrangement (Figure 1) was set to 100 mm for bending strength measurements and 10 mm for shear strength measurements, respectively. All tests were carried out at room temperature. The different types of developed composites are presented in Figure 2a–c.

**Figure 3:**
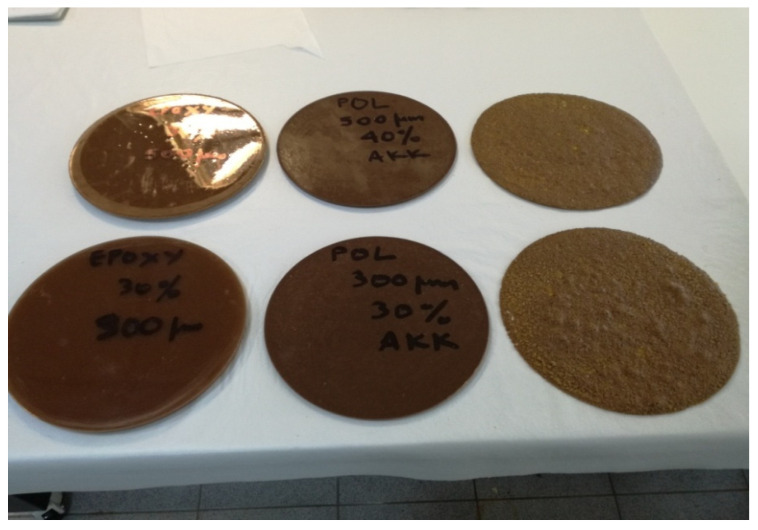
Composite specimens for thermal insulation efficiency testing [From left to right Epoxy Resin Composites (ERC), Unsaturated Polyester Composites (UPC), and Novolac Composites (NC) specimens].

On the other hand, composites’ thermo-insulating efficiency study was performed via the evaluation of thermal conductivity coefficient λ within the context of ASTM C 177 [66]. The specimens manufactured for the purposes of thermal efficiency study are shown in Figure 3.

Calculations of composites’ bending (σ_b_) and shear strength (τ_b_) were performed using the following equations:

(1) $$\sigma_{b}=\frac{3\times P_{\max}\times L_{s}}{2\;\times \;b\;\times {\;d}^{2}}$$

(2) $$\tau_{b}=\frac{0.75{\;\times \;P}_{\max}}{b\;\times \;d}$$

where P_max_ is the maximum load applied at specimen’s failure (N); Ls is test length (m); b is specimen’s width (m); and d is specimen’s thickness (m).

The error involved in all flexural and shear strength measurements was ±7%.

Similarly, thermal insulating efficiency of produced composites was made by determination of the thermal conductivity coefficient λ, according to ASTM C 177 standard using specimens of appropriate shape and dimensions, which was evaluated by the following equation:

(3) $$\lambda =\frac{\Phi \times S_{m}}{2A\left(\Theta_{wm}-\Theta_{cm}\right)}$$

where Φ is the capacity resistance of the heating surface, Sm is the composites’ average thickness (m), A is the composites’ average surface area (m^2^), Θ*wm* is the composites’ warm surfaces average temperature (°K), and Θ*cm* is the composites’ cold surfaces average temperature (°K).

The percentage error involved in the measurement of thermal properties was ±5%.

### 2.4. Characterization Methods

The surface structural evaluation and elemental analysis were performed via scanning electron microscopy (SEM), energy dispersive X-ray (EDX), and X-ray diffraction (XRD). SEM measurements were carried out using a Hitachi’s TM3030 Plus (Hitachi, Tokyo, Japan) scanning electron microscope equipped with a QUANTAX 70 (Hitachi, Tokyo, Japan) energy dispersive X-ray spectrometer (EDS). Finally, characterization of the produced PMCs and the C&D powders used as fillers in the preparation of the resulting specimens was performed using a Siemens D5000 Diffractometer (Siemens, Karlsruhe, Germany), equipped with a Cu Ka source (*λ* = 0.15406 nm). The scanning range was set from 5° to 70° with a step of 0.04° and a time interval of 1 sec per step. The crystalline phase contents of the powder samples were determined by X-ray diffraction. The crystallite sizes (d) of the resulting composites as well as for the different filling powders were calculated by Debye–Scherrer’s [67] equation as follows:

(4) $$d=\frac{K\times \lambda }{\beta \times \cos\theta }$$

where *Κ* = 1.84 is the Debye–Scherrer’s constant, *λ* = 0.15406 nm is the wavelength of X-ray radiation of the equipment used, θ is the Bragg diffraction angle (°), and β is the full width half maximum (FHWM) of the highest diffraction peak.

## 3. Results and Discussion

### 3.1. Mechanical Characterization

The bending and shear strength of resulting materials were determined using, as mentioned previously, the three-point method. Test results are presented in Table 3. Novolac matrix composites loaded with C&D wastes at percentages of 40% and 50% *w*/*w* were not experimentally examined because manufacturing specimens under these specific resin/ filler proportional characteristics was not possible. All the categories of PMCs studied exhibited a brittle behavior, as do most thermosetting materials [62,68]. In parallel, these materials were characterized by a significant reduction in mechanical performance, remaining though in acceptable levels of mechanical strengths in comparison to common building materials [69,70,71]. The lowering of these materials’ mechanical performance came as a consequence of C&D waste powder inclusion within the polymer matrix. All tested samples presented identical fracture patterns with clear and abrupt breaking at ultimate loading (failure point).

As it can be observed, PMCs mechanical strength decreased, in respect to specific manufacturing characteristics of the resulting materials such as (i) the *w*/*w* percentage of embedding filler, (ii) the embedding filler’s grain size, and (iii) the kind of resin used as matrix. Analytically, the flexural strength was measured to be approximately three times lower (60.03 MPa for 30% *w*/*w*, 300 μm specimen) to seven times lower (24.42 MPa for 50% *w*/*w*, 500 μm specimen) for ERCs in comparison to pure ER materials (166.87 MPa). Accordingly, flexural strength was two times lower (35.61 MPa for 40% *w*/*w* 300 μm specimen) to three times lower (27.47 MPa for 50% *w*/*w*, 500 μm specimen) for UPC specimens compared to these made of pure UP (75.30 MPa) and relatively lower (21.79 MPa for 30% *w*/*w* for both 300 μm and 500 μm specimens) for N-based composites in comparison to pure N specimens (26.80 MPa). 

Similarly, ERCs’ shear strength was measured to be approximately two times lower (7.54 MPa for 30% *w*/*w*, 300 μm specimen) to seven times lower (2.05 MPa 50% *w*/*w*, 500 μm) in comparison to shear values measured for pure ER specimens (13.8 MPa), approximately four times lower (3.75 MPa 40% *w*/*w*, 300 μm) to six times lower (2.50 MPa for 50% *w*/*w*, 500 μm specimen) for UP composites compared to these measured for pure UP specimens (13.95 MPa) and slightly lower in magnitude for NCs’ (1.26 MPa for 30% *w*/*w*, 300 μm and 1.21 MPa for 30% *w*/*w* 500 μm, respectively) in relation to these made using neat novolac (1.81 MPa). UP specimens that were filled using C&D waste at 40% *w*/*w* presented slightly improved flexural (35.61 MPa for 300 μm and 34.6 MPa for 500 μm specimen) and shear (4.18 MPa for 300 μm and 3.87 MPa for 500 μm specimen) strengths compared to those containing 30%, in contradiction to ERC and NC in which the increase of embedding substance in the composite from 30% *w*/*w* to 40% *w*/*w*, resulted in materials of lower mechanical strength as shown from the results. 

Generally, according to the test results, ERCs were the optimum materials in terms of mechanical properties, followed by UPCs and NCs, respectively. More specifically, 300 μm 30% *w*/*w* containing ERCs, demonstrated better mechanical performance, in terms of flexural and shear strength amongst all others, followed by 300 μm 40% *w*/*w* filler containing UPCs and NCs, exhibiting approximately two times higher flexural strength (60.03 MPa) compared to UPCs (35.61 MPa) and three times higher compared to NCs (21.79 MPa), respectively. In the same way, the above-discussed composite materials exhibited two times higher shear strength (7.54 MPa) compared to UPCs (3.72 MPa) and six times higher shear strength in comparison to NCs (1.26 MPa), respectively.

Encapsulation of larger grain size filler and maintaining filler *w*/*w* concentration constant (i.e., 30%) resulted in materials with differentiated mechanical characteristics. More specifically, 500 μm ERCs were characterized by flexural properties that were almost identical (34.59 MPa) to those of 500 μm UPCs (33.58 MPa) and approximately 1.5 times higher compared to 500 μm NCs (21.79 MPa), while their shear strength was measured to be approximately two times higher (4.18 MPa) compared to that of 500 μm UPCs (2.81 MPa) and three times higher in comparison to 500 μm NCs (1.21 MPa). As shown in Table 3, epoxy and polyester composites were characterized by flexural strengths that were found to be almost six and up to twelve times higher compared to their corresponding shear strengths. 

On the other hand, those manufactured using N resin demonstrated about 20 times higher flexural strength values compared to the corresponding shear strength values of these materials. Finally, another important remark was that the flexural and shear performances of ERCs loaded in proportions of 30% and 40% were significantly reduced, once larger granular size filler was used, whereas all other PMCs examined were characterized by small and, in some cases, insignificant changes in mechanical properties.

### 3.2. Thermal Insulation Efficiency

PMCs’ thermal insulation performance results are presented in Table 4. As it can be observed, the inclusion of C&D wastes micro-powder resulted in materials with improved thermo-insulating efficiency compared to that exhibited by pure resin specimens. The increase of encapsulated filler’s (*w*/*w*) quantity and the utilization of lower granular size pulverized filler within the composites’ matrix led to further improvement of the thermal conductivity coefficient λ and therefore enhancement of these materials’ thermal insulation performance. Ν and UP composites exhibited better thermal insulation properties compared to these manufactured using ER. This came as a result of the structural peculiarity of these specimens, which has been developed during the thermal processing stage (curing) as indicated via SEM analysis and discussed in Section 3.3. However, the advantage of UPC and NC in terms of thermo-insulating properties is also associated with the good insulating characteristics of polyester and novolac resins in general. As it is observed from the following results, UP composites’ thermal conductivity coefficient λ was slightly increased, remaining, however, in a close range of λ values to these exhibited by neat UP materials.

### 3.3. SEM, EDX, and XRD Characterization

**Figure 4:**
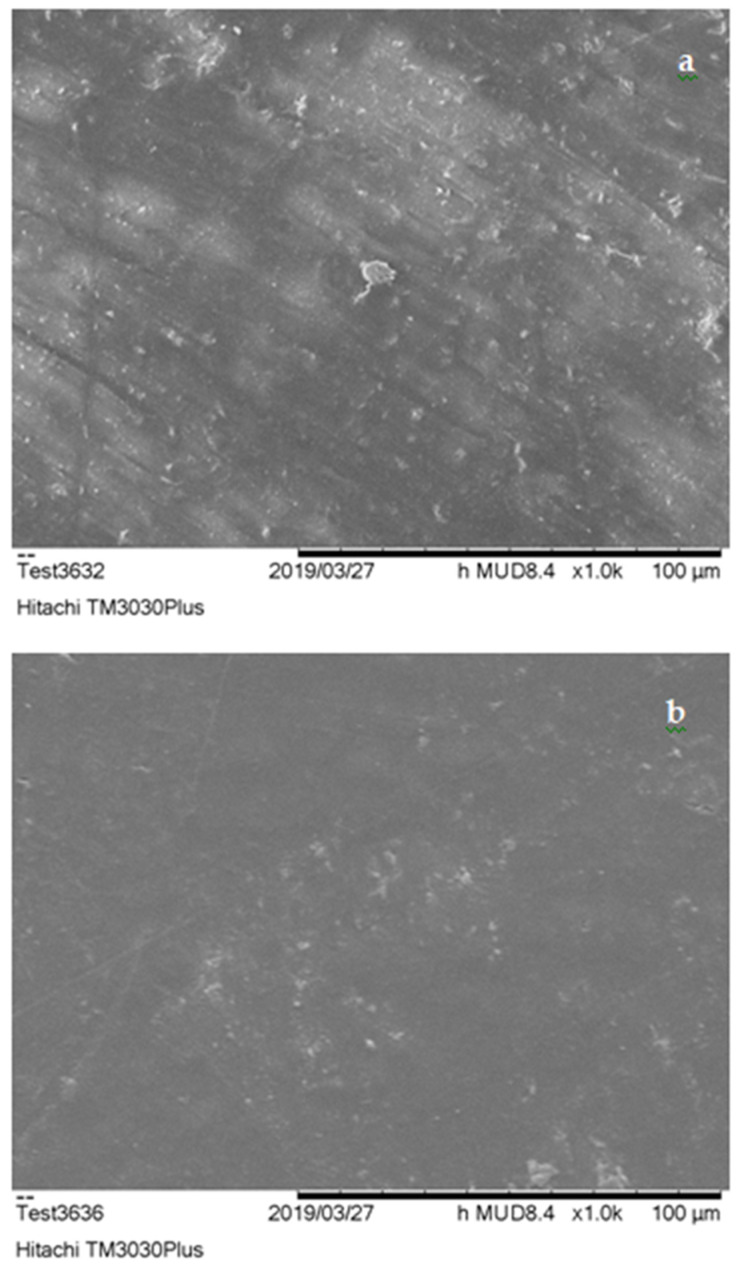
ERCs SEM imaging; (**a**) 30% of 500 μm construction and demolition (C&D) waste (*w*/*w*), magnified ×1000 and (**b**) 30% of 300 μm C&D waste (*w*/*w*), magnified ×1000.

**Figure 5:**
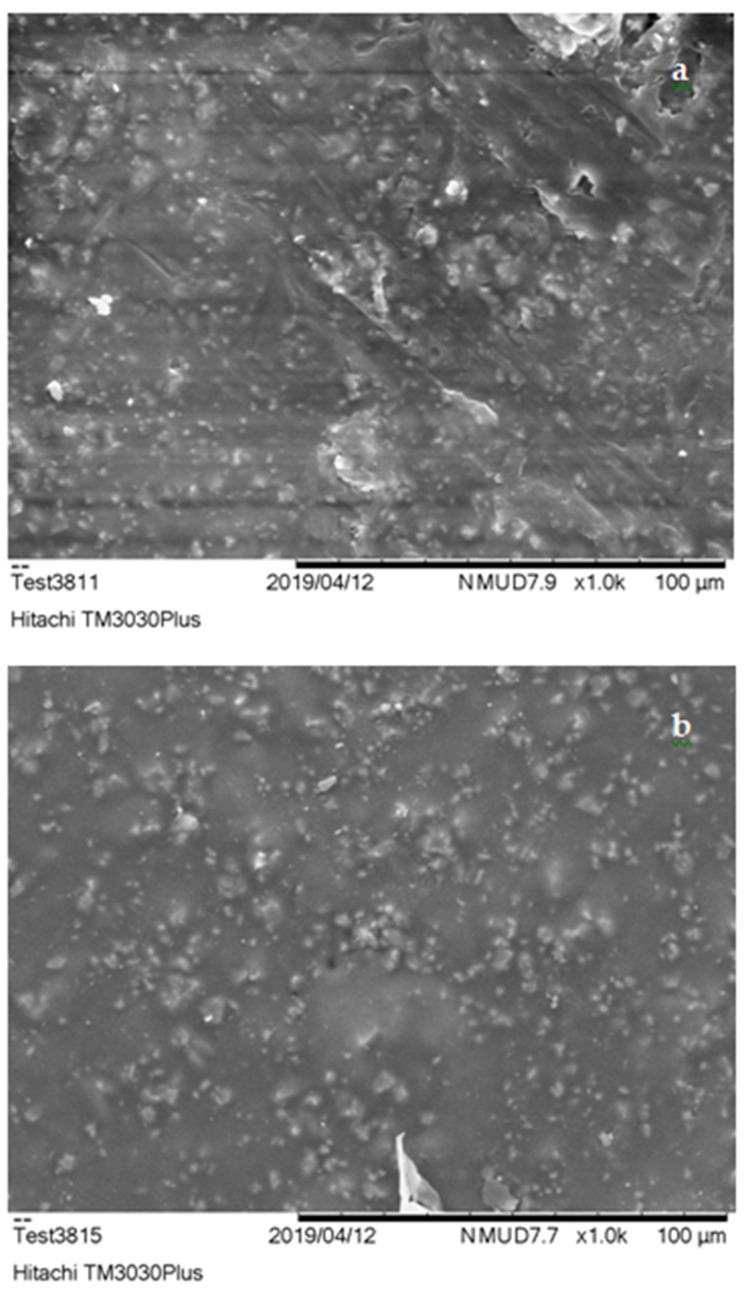
UPCs SEM imaging; (**a**) 40% of 500 μm C&D waste (*w*/*w*); C&D waste magnified ×1000 and (**b**) 40% 300 μm C&D waste (*w*/*w*); C&D waste magnified ×1000.

**Figure 6:**
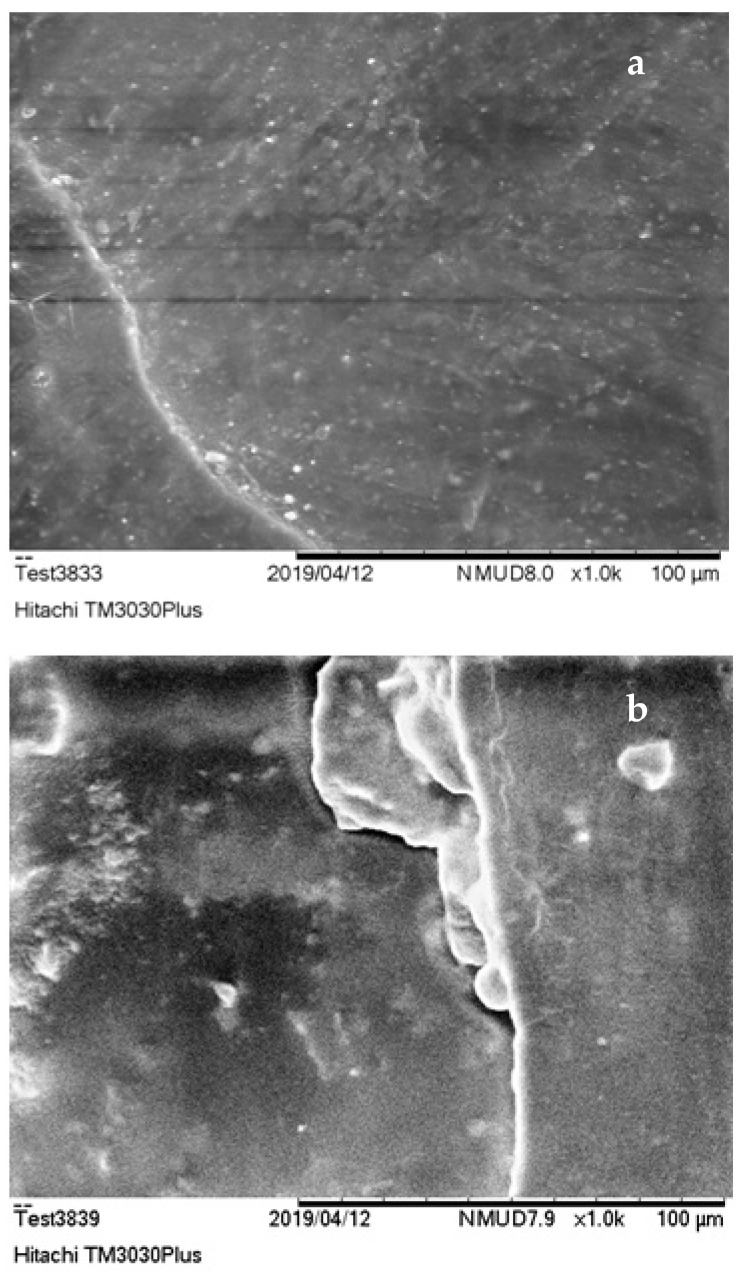
NCs SEM imaging; (**a**) 30% of 500 μm C&D waste (*w*/*w*), magnified ×1000 and (**b**) 30% of 300 μm C&D waste (*w*/*w*), magnified ×1000.

SEM characterization results are presented in Figure 4, Figure 5 and Figure 6. According to these, the dispersion of filling powder in ER composites (Figure 4a,b) was quite satisfactory with sparse agglomerations within the specimens’ matrix. The dispersion of embedded additive was improved, while smaller size embedding filler was used, resulting in the production of PMCs with upgraded mechanical strength. 

On the other hand, UPCs, (Figure 5a,b) exhibited better dispersion and minimal agglomerate formations in comparison to ERCs. The use of smaller size filler resulted again in the improvement of its distribution in the polymer matrix leading in parallel to the enhancement of these materials’ mechanical properties. The improved level of dispersion of filler exhibited in UPCs is a result of the reduced time required for the hardening process of the unsaturated polyester matrix to take place (approximately 45–55 min according to Table 1). As far as the improvement in the dispersion of embedded substance of NCs is concerned, this is strongly related to the granular nature that characterizes both novolac resin and filling material used, which enabled their better mixing during the preparation of the composite specimens.

As shown in the SEM images, UP and N specimens (Figure 5 and Figure 6) were characterized by voids shaped within the matrix. These came as a result of volatile gas evolution that occurred during the composites curing process [72]. The presence of these voids affected the structural coherence of specimens and is in fact responsible for the lower flexural and shear performance of these materials. The entrapment of air produced (i.e., voids) during PMCs thermal processing stage in the form of bubbles led to the enhancement of their thermo-insulating properties. This thermal insulation efficiency was further improved in composite specimens that embedded filler of smaller grain size. Due to the lower size of filling powder, its distribution within the polymer matrix was improved, allowing the further improvement of these materials’ thermo-insulating performance.

EDX analysis indicated as expected the presence of carbon and oxygen, the main constituents of the matrix of manufactured composites, with carbon holding the highest proportion amongst all detected elements (Table 5). Carbonate acids of silicon (Si) and calcium (Ca) were also detected within the composites. In addition, carbonate acids of magnesium (Mg) were detected through the EDX analysis conducted on samples of the filling powders.

Additionally, X-ray diffraction indicated calcite (CaCO_3_) and quartz (SiO_2_) corresponding to database patterns PDF 72-1937, PDF 047-1144, and PDF 083-2187 [73,74,75], respectively, as the characteristic crystalline phases contained in the 300 μm and 500 μm fillers (Table 6). Both the embedding powders exhibited similar behavior and therefore identical XRD spectra.

**Figure 7:**
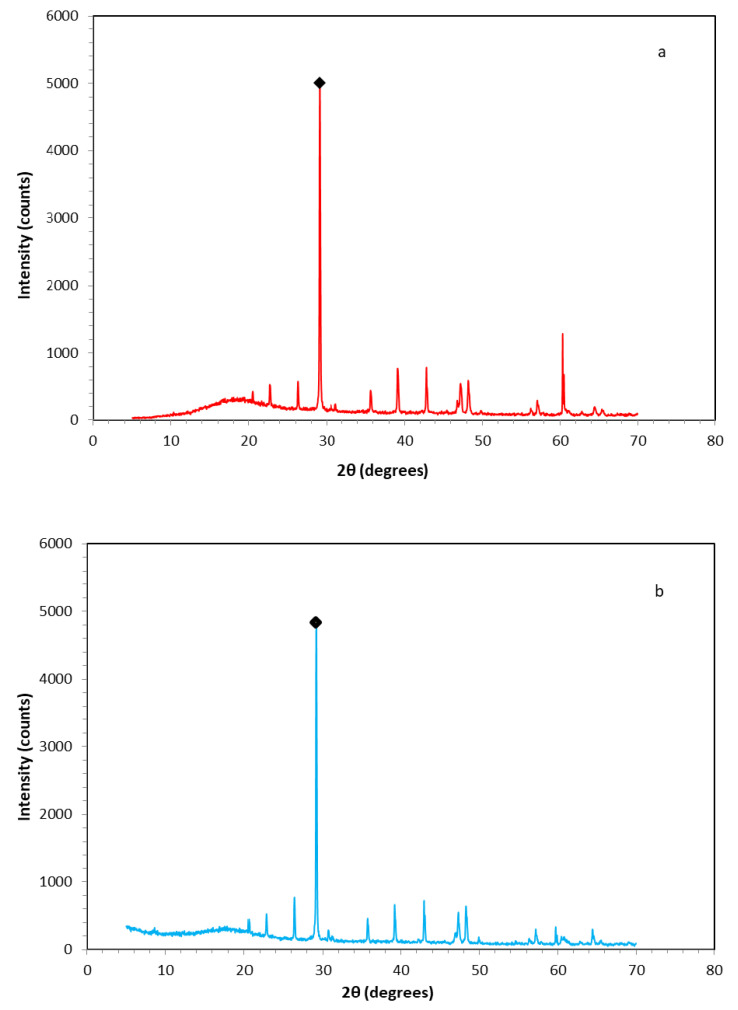
X-ray diffractograms of produced filling powders; (**a**) C&D waste (CDW) powder of 300 μm and (**b**) CDW powder of 500 μm. (The black square mark indicates the 2θ = 29.1°, which is assigned to the calcite crystalline phase).

The XRD peaks of the above samples were observed within the range (2θ) of 20° and 66°, as can be observed through the diffractograms shown in Figure 7. The characteristic peak value of 2θ = 29.1°, which is marked with a black square-shape in the previously mentioned diagram, was assigned to the calcite phase plane (104), and it appears to be dominant for both the powder samples examined via the X-ray diffraction process.

**Figure 8:**
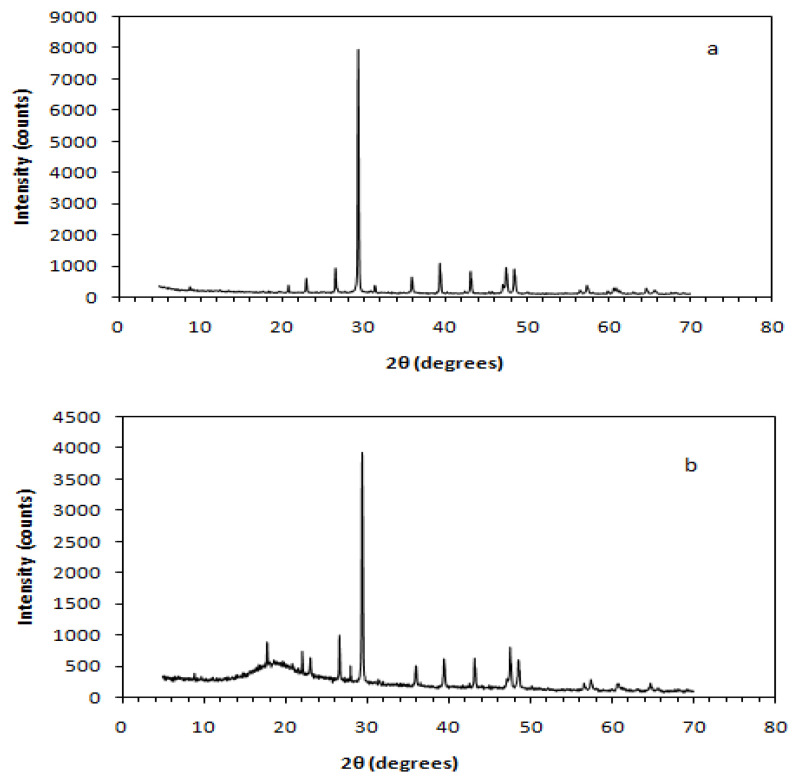
X-ray diffractograms of epoxy resin matrix composites (**a**) 300 μm composites and (**b**) 500 μm composites.

**Figure 9:**
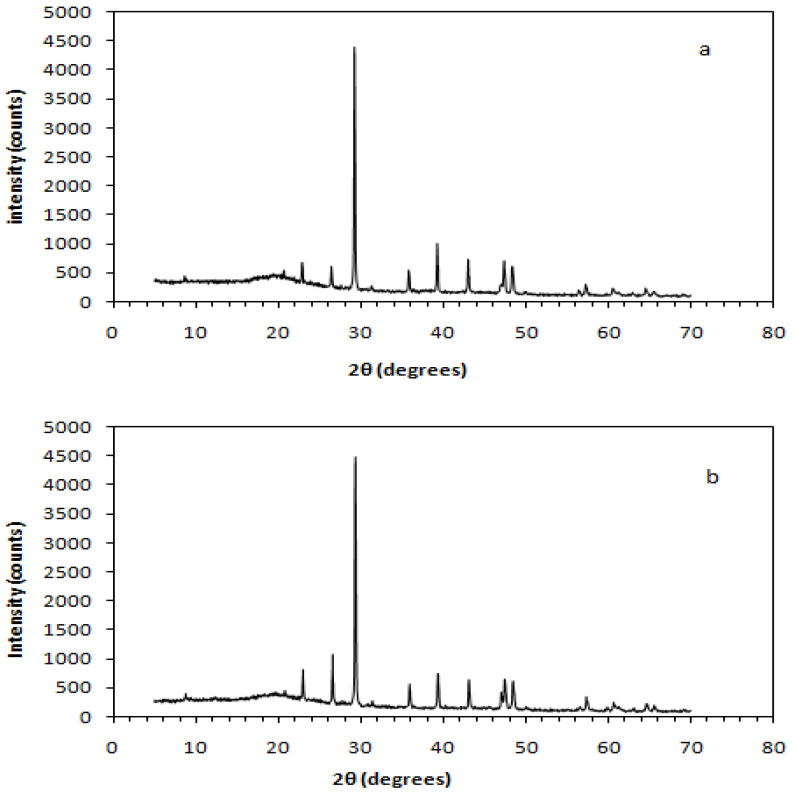
X-ray diffractograms of polyester resin matrix composites (**a**) 300 μm composites and (**b**) 500 μm composites.

**Figure 10:**
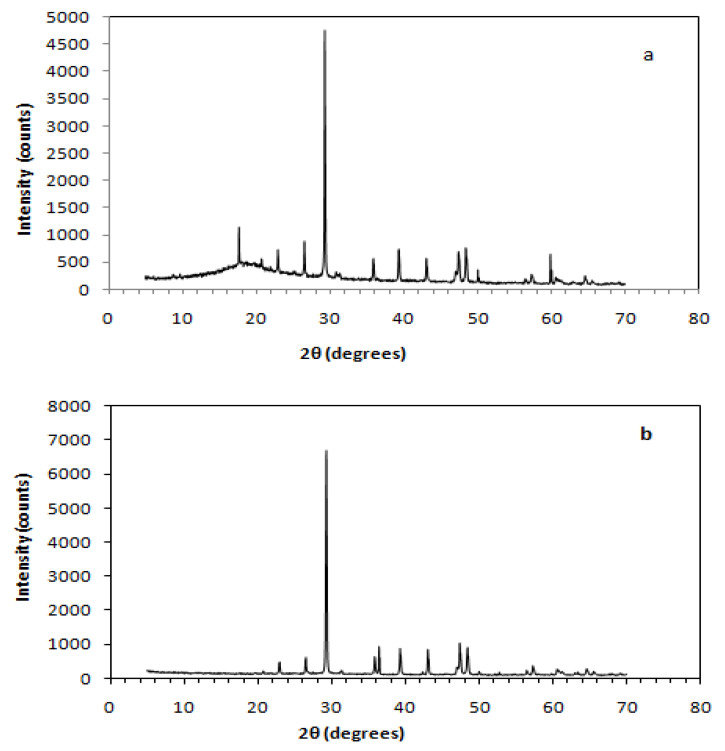
X-ray diffractograms of novolac resin matrix composites (**a**) 300 μm composites and (**b**) 500 μm composites.

Figure 8, Figure 9 and Figure 10 depict XRD spectra of the dominating (in terms of flexural and shear strength) epoxy, unsaturated polyester, and novolac matrix composites, respectively. Each one of these figures is characterized by the presence of two distinct phases—the crystal phase, which is attributed to the crystallinity of the embedded (C&D waste) powder, and the amorphous phase, which is related to the polymer resin used as matrix. 

The sharp peaks appearing in the graphs are assigned to the crystal phase of the composite, whereas the low peaks are assigned to the amorphous phase appearing in low 2θ value ranges usually under 30°. More precisely, the 2θ peak value of the amorphous phase in ER–CDW30-500 μm composites is approximated at 17.74°, while in ER–CDW30-300 μm the amorphous phase practically “disappears” as a result of the increased intensity of the crystal phase peaks (Figure 8a,b). Novolac composites exhibited similar behavior with the amorphous phase of N–CDW30-300 μm located approximately at 17.71°, while in N–CDW30-500 μm, the increased intensity of the crystal phase peaks characterizing this material led again to the disappearance of the low-intensity amorphous phase peaks (Figure 10a,b).

Finally, the amorphous phase peaks of UP–CDW40-500 μm and UP–CDW40-300 μm were approximated at 2θ = 19.95° (Figure 9a,b). The amorphous phase 2θ values measured for ER, UP, and N composites fall within a corresponding range of values that are recorded and referred to in the literature [76,77,78,79,80,81,82,83,84,85].

The crystallite sizes (d) of the resulting composites and the filling powders at the highest diffraction peak angles, recorded through the XRD characterization, are presented in Table 7. As shown below, the crystallites of 500 μm and 300 μm fillers were found to be identical in size. On the other hand, the crystallites that were shaped within the ER, UP, and N composites under study exhibited size variations in respect to the polymer resin used to form the matrix. Changes in the size of crystallites were also observed between the composites made using the same type of polymer as matrix when different size filler was used, with that magnitude (crystallite size) being reduced once 500 μm powder was used as filler instead of 300 μm in all the PMC categories examined.

## 4. Conclusions

Thermoset polymer composites of epoxy and unsaturated polyester that were purchased from the market and laboratory synthesized phenol-formaldehyde (novolac), embedding pulverized C&D waste were developed and studied. The mechanical, thermal, and structural characterizations of these materials were made by means of bending and shear testing, SEM, EDX, and XRD, respectively. The presence of C&D waste limited down the mechanical performance, which was maintained however, in adequate levels. On the contrary, the thermal insulation efficiency was improved after the incorporation of C&D filler in most of the PMCs examined.

## Figures and Tables

**Table 1:** Epoxy and unsaturated polyester resins technical specifications.

Resin	Viscosity [Pa s]	Density [g/cm^3^]	Pot Life [min]	Hardening Time [min]	A:B Mixture Analogy (*w*/*w*)
Epoxol 2874	1.22	1.09	35–45	240	100:58
PE6/TC	0.55–0.65	1.2	20–25	45–55	100:2
Laboratory made Novolac (powder)	-	0.9	-	160	7:2 (HEXA as hardener)

**Table 2:** Composites manufactured for mechanical and thermal characterization.

PMC Name	Filler (% *w*/*w*)	Resin (% *w*/*w*)	Comment
ER-100	0	100	Mech./ thermal properties testing
ER–CDW30-500μm	30	70	Mech./ thermal properties testing
ER–CDW40-500μm	40	60	Mech./ thermal properties testing
ER–CDW50-500μm	50	50	Mechanical properties testing
ER–CDW30-300μm	30	70	Mech./ thermal properties testing
ER–CDW40-300μm	40	60	Mech./ thermal properties testing
ER–CDW50-300μm	50	50	Mech./ thermal properties testing
UP-100	0	100	Mech./ thermal properties testing
UP–CDW30-500μm	30	70	Mech./ thermal properties testing
UP–CDW40-500μm	40	60	Mech./ thermal properties testing
UP–CDW50-500μm	50	50	Mechanical properties testing
UP–CDW30-300μm	30	70	Mech./ thermal properties testing
UP-CDW40-300μm	40	60	Mech./ thermal properties testing
UP–CDW50-300μm	50	50	Mechanical properties testing
N-100	0	100	Mech./ thermal properties testing
N–CDW30-500μm	30	70	Mechanical/ thermal properties testing
N–CDW30-300μm	30	70	Mech./ thermal properties testing

**Table 3:** Comparative analysis of flexural and shear strength of polymer composites under study.

PMC Name	Filler (% *w*/*w*)	Resin (% *w*/*w*)	Flex. Strength (MPa)	Shear Strength (MPa)
ER-100	0	100	166.87	13.80
ER–CDW30-300μm	30	70	60.03	7.54
ER–CDW40-300μm	40	60	39.68	3.57
ER–CDW50-300μm	50	50	26.45	2.66
ER–CDW30-500μm	30	70	34.59	3.72
ER–CDW40-500μm	40	60	25.43	3.42
ER–CDW50-500μm	50	50	24.42	2.05
UP-100	0	100	75.30	13.95
UP–CDW30-300μm	30	70	34.59	3.72
UP–CDW40-300μm	40	60	35.61	4.18
UP–CDW50-300μm	50	50	30.25	2.66
UP–CDW30-500μm	30	70	33.58	2.81
UP–CDW40-500μm	40	60	34.61	3.87
UP–CDW50-500μm	50	50	27.47	2.50
N-100	0	100	26.80	1.81
N–CDW30-300μm	30	70	21.79	1.26
N–CDW30-500μm	30	70	21.79	1.21

**Table 4:** Epoxy, unsaturated polyester, and novolac resin composites thermal conductivity coefficient λ.

Composite	C&D (% *w*/*w*)	Resin (% *w*/*w*)	Thermal Conductivity Coefficient, *λ* [W/m∙K]
ER-100	0	100	1.20
ER–CDW30-500μm	30	70	0.70
ER–CDW40-500μm	40	60	0.64
ER–CDW30-300μm	30	70	1.02
ER–CDW40-300μm	40	60	0.53
UP-100	0	100	0.27
UP–CDW30-500μm	30	70	0.59
UP–CDW40-500μm	40	60	0.46
UP–CDW30-300μm	30	70	0.63
UP–CDW40-300μm	40	60	0.39
N-100	0	100	0.72
N–CDW30-500μm	30	70	0.42
N–CDW30-300μm	30	70	0.36

**Table 5:** Polymer matrix composites and embedded fillers elemental analysis.

PMC Name	C (wt.%)	O (wt.%)	Ca (wt.%)	Si (wt.%)	Al (wt.%)	Mg (wt.%)	Total
ER–CDW30-500μm	77.27	18.18	3.73	0.81	-	-	**100.00**
ER–CDW30-300μm	73.75	19.03	5.55	1.66	-	-	**100.00**
UP–CDW40-50 μm	69.08	26.24	3.69	0.99	-	-	**100.00**
UP–CDW40-300μm	60.57	31.21	7.01	1.21	-	-	**100.00**
N–CDW30-500μm	72.01	23.75	3.62	0.62	-	-	**100.00**
N–CDW30-300μm	81.94	16.12	1.60	0.35	-	-	**100.00**
**Type of filler**							
500 μm CDW filler	11.09	46.75	35.27	4.53	2.08	0.37	**100.00**
300 μm CDW filler	12.60	51.73	26.81	5.55	2.75	0.57	**100.00**

**Table 6:** Identified crystalline patterns contained in the filling powders of 500 μm and 300 μm used in composites manufacturing.

Composite	Crystalline Name	Formula	Pattern PDF
500 μm CDW filler	Calcite	CaCO_3_	72-1937
-	Quartz	SiO_2_	01-083-2187
300 μm CDW filler	Calcite	CaCO_3_	72-1937
-	Quartz	SiO_2_	01-047-1144

**Table 7:** Crystallite sizes of fillers and resulting composites.

Composite	Crystallite Size (nm)
300μm CDW filler	116.65
500 μm CDW filler	116.65
ER–CDW30-500 μm	107.71
ER–CDW30-300 μm	114.43
UP–CDW40-500 μm	107.70
UP–CDW40-300 μm	110.44
N–CDW30-50 0μm	99.96
N–CDW30-300 μm	122.54

## Data Availability

The data presented in this study are available on request from the corresponding author.
